# Perceptions of Young Patients During Digital and Conventional Impression-Taking: A Comparative Semi-experimental Randomized Study

**DOI:** 10.7759/cureus.93729

**Published:** 2025-10-02

**Authors:** Loubna Benkirane, Hakima Aghoutan, Nouhaila Alaoui, Fatiha Atifi, Farid El Quars, Samira Elarabi

**Affiliations:** 1 Pediatric Dentistry, Faculty of Dental Medicine, Hassan II University of Casablanca, Casablanca, MAR; 2 Orthodontics and Dentofacial Orthopedics, Faculty of Dental Medicine, Hassan II University of Casablanca, Casablanca, MAR; 3 Dentofacial Orthopedics, Faculty of Dental Medicine, Hassan II University of Casablanca, Casablanca, MAR

**Keywords:** conventional impression, digital impressions, patient comfort, patient perceptions, pediatric dentistry

## Abstract

Introduction

Optical impressions have revolutionized dentistry, but their use in children under 10 years old remains underexplored due to age-specific challenges.

Objective

This study aimed to evaluate levels of comfort, cooperation, preferences, and time required for conventional versus digital impressions in children consulting at the Dental Consultation and Treatment Center in Casablanca.

Methods

This semi-experimental, randomized crossover study compared conventional alginate impression and digital impression in 40 children and adolescents aged 6-14 years. Participants underwent both techniques in a random order, with a seven-day interval between sessions. Data analysis included descriptive statistics and inferential tests (Student's t-test, ANOVA, χ2 test, Fisher's test, McNemar's test, Mann-Whitney U test, and Kruskal-Wallis test) to study associations between sociodemographic characteristics and patient preferences and behaviors regarding dental impressions.

Results

The mean age of participants was 9.3±2.25 years. Digital impressions were preferred by 62.5% of children, who found them quicker and more comfortable. Around 22.5% of children experienced breathing discomfort and a gag reflex with conventional impressions. About 60% of children found conventional impressions tiring, compared to 17.5% for digital impressions. Around 62.5% of children would choose digital impressions for future procedures, reflecting Gen Z's affinity for interactive experiences.

Discussion

This study demonstrates that digital impressions with Trios 3 (3Shape, Copenhagen, Denmark) are perceived as more comfortable and faster than conventional alginate impressions among children aged 6-14 at the Casablanca center, aligning with prior pediatric research that reports similar advantages of intraoral scanners over traditional methods while noting no major differences in anxiety or behavior. However, the results should be interpreted within the context of a single scanner model and a crossover design. Further multicenter studies with diverse devices and larger samples are warranted to confirm these findings.

Conclusion

Digital impressions have shown superiority, even among younger patients. Nevertheless, their adoption is hindered by high costs, a steep learning curve, sensitivity to hypersalivation, and head movement. Conventional impressions remain relevant for specific indications, and prior child preparation is crucial for a successful outcome.

## Introduction

In dentistry, precision in reproducing oral structures is crucial for both adults and children. Alginate impressions are widely used because they are simple, inexpensive, and quick to take. However, this method has several drawbacks, including patient discomfort, dimensional instability, model fragility, and storage difficulties [1]. Digital technologies have led to the development of intraoral scanners, which offer a potentially superior alternative with advantages such as accuracy, 3D previsualization, reduced distortion, unlimited digital storage, and increased patient comfort [2,3].

Since the introduction of the first intraoral scanner in 1999 (OrthoCAD, Version 1, Cadent Technologies Inc., Carlstadt, New Jersey, United States) [4], several devices have been marketed, and numerous studies have compared their performance to that of conventional impressions in adults [5-9]. However, the results remain contradictory due to the diversity of methodologies and devices used [10-12]. Few studies have focused on the pediatric population, particularly children under the age of 10 [13-18], who make up a large proportion of patients requiring orthodontic treatment and are often more likely to experience discomfort or fear at the dentist. The idea of replacing conventional impressions with digital impressions is therefore particularly interesting for this population. However, the behavioral characteristics of children and the presence of smaller oral structures can compromise both conventional and digital impression procedures [19]. While digital scanners are increasingly popular in adult practice, their acceptability and comfort in children remain poorly evaluated, particularly in African or national contexts. Our study aims to compare children's experiences with conventional and digital impressions and to evaluate their perception of comfort and tolerance, time, and preference, with the null hypothesis that there is no significant difference between these methods in terms of perception in children.

## Materials and methods

This is a semi-experimental, randomized, crossover study, in accordance with the Consolidated Standards of Reporting Trials (CONSORT) guidelines for clinical trials [20]. The order in which the impression techniques were assigned was determined by randomization, without the operator's knowledge. Although recruitment was not entirely random, each participant underwent both impression techniques in random order, separated by a seven-day interval to limit bias related to memory or fatigue.

Ethical approval

The study was approved by the Pedagogic and Research Conduct Committee of the Faculty of Dental Medicine at Hassan II University of Casablanca (approval number: FMDC-pr2c/29.30-2025) and conducted in accordance with the requirements of the Declaration of Helsinki for biomedical research. After taking the necessary steps to obtain patient access and recruitment authorizations, as well as to acquire the required materials and equipment, all parents or legal guardians were informed of the study's objectives and procedures and gave their written informed consent.

Study population and sample size calculation

Participants, aged 6-14 years, were recruited from among children and adolescents awaiting consultation or treatment in the Department of Pediatric Dentistry or Orthodontics at the Dental Consultation and Treatment Center in Casablanca. The sample size was determined based on data from the literature, assuming a 50% difference in preference between the two methods, with an alpha error of 0.05 and a power of 80%. Thus, 40 subjects were required. During each session, five patients were included.

To be included in the study, they had to be aged between six and 14 years old, have no experience of dental impression techniques or intraoral scanning, and have satisfactory cooperation (psychological profile determined by subjective assessment).

Interventions

A preliminary phase was conducted with 20 children to test the feasibility and comprehensibility of the questionnaires. The order of interventions was assigned randomly. During this stage, we used empty impression trays to simulate conventional impressions, while a curing light was used to imitate digital impressions. Each of these methods was applied according to a pre-determined random selection.

This phase allowed us to experiment with the children's perception of the two techniques, familiarize ourselves with the study conditions, test and validate the questions, and anticipate any limitations and difficulties that might arise during the main phase of the study.

Conventional impressions were taken using alginate (Cavex Ca37 Fast Set Dust-Free, Cavex Holland BV, Haarlem, Netherlands) and plastic impression trays, mixed manually with drinking water according to the manufacturer's instructions. Digital impressions were obtained using the Trios 3 intraoral scanner (3Shape, Copenhagen, Denmark) after drying the tooth surfaces, starting systematically with the mandibular arch.

An experienced operator took all impressions, while two doctoral students were responsible for randomizing patients, filling out forms, and assigning tasks (mixing and taking impressions or managing timing).

Each type of impression was taken during separate sessions, one week apart, to minimize any influence on the perception of comfort. Patients who could not be reached were replaced by others in order to maintain the sample size.

Statistical analysis

Data analysis was performed using jamovi (Version 2.4) (The jamovi project (2023) [Computer Software]. Retrieved from https://www.jamovi.org) and included descriptive and analytical statistics. To examine associations between sociodemographic characteristics and patient preferences or behaviors regarding dental impressions, statistical tests such as the χ2 test, Fisher's exact test, and ANOVA were used. Paired sample tests were used to compare intra-individual data in the cross-over context, with Student's t-test for quantitative variables and McNemar's test for qualitative variables. The impact of age, gender, and order of impressions on the comparative perception between the two techniques was assessed using the Mann-Whitney U test for gender and the Kruskal-Wallis test for age groups. A 95% confidence interval was chosen with a significance threshold set at 0.05.

## Results

A total of 40 children participated in the study, which took place from October 2023 to February 2024. Seven children were replaced due to their absence during the second impression-taking session. The sample included 24 boys with a mean age of 9.16±2.25 years and 16 girls with a mean age of 9.38±2.32 years. The overall mean age was 9.3±2.25 years. No significant difference was found between the ages of boys and girls (Student's t-test). One patient had difficulty breathing when taking the conventional mandibular impression compared to eight for the conventional maxillary impression. The difference between the two was significant, with a p-value of 0.008.

A significant association was found for the gag reflex between digital and conventional maxillary impressions (p=0.002) and between conventional maxillary and mandibular impressions (p=0.002). Only one patient vomited during conventional maxillary impression-taking (Table 1).

**Table 1:** Distribution of participants according to the parameters related to the level of comfort compared between the two impression techniques Conv: conventional; Dig: digital; max: maxillary; mand: mandibular; N: number

	Conv max N%	Dig max N%	Conv mand N%	Dig mand N%	Comparison	McNemar's test
The child closed their eyes	26 (65%)	33 (82.5%)	18 (45%)	16 (40%)	Conv max vs. dig max	0.071
					Conv max vs. conv mand	0.021
					Conv mand vs. dig mand	0.617
The child moved their hands or feet	19 (47.5%)	21 (52.5%)	17 (42.5%)	14 (35%)	Conv max vs. dig max	0.637
					Conv max vs. conv mand	0.480
					Conv mand vs. dig mand	0.366
The child had difficulty breathing	8 (20%)	0 (0%)	1 (2.5%)	0 (0%)	Conv max vs. conv mand	0.008
The child had a gag reflex	11 (27.5%)	1 (2.5%)	1 (2.5%)	0 (0%)	Conv max vs. dig max	0.002
					Conv max vs. conv mand	0.002
The child vomited	1 (2.5%)	0 (0%)	0 (0%)	0 (0%)	-	-
The child cried	2 (5%)	0 (0%)	1 (2.5%)	1 (2.5%)	-	-

Approximately 22.5% of patients (mean age: 9.22±2.59 years) experienced discomfort during digital impression-taking, compared with 40% (mean age: 9.63±2.53 years) during conventional impression-taking. There was no significant difference (Table 2).

**Table 2:** Distribution of participants according to their level of cooperation by age and sex for each impression technique

Impression technique		Behavior						Anxiety				
		Negative			Positive	Definitely positive	p	Relaxed	Uncomfortable	Tense	Reluctant	p
Digital	N(%)	2 (5%)			29 (72.5%)	9 (22.5%)	-	26 (65%)	9 (22.5)	4 (10%)	1 (2.5%)	-
	Age (years)	7.00±1.41			9.34±2.11	9.61±2.78	0.268	9.29±2.11	9.22±2.59	9.75±3.30	8.00	0.941
	Sex	F		0	10	6	0.140	11	4	1	0	0.759
		M		2	19	3		15	5	3	1	
Conventional	N(%)	0 (0%)			33 (82.5%)	7 (17.5%)	0.472	18 (45%)	16 (40%)	6 (15%)	0 (0%)	0.504
	Age (years)	-			9.39±2.27	8.79±2.38		9.31±2.11	9.63±2.53	8.33±2.07	-	
	Sex	F	0		12	4	0.407	10	5	1	0	0.151
		M	0		21	3		8	11	5	0	

The digital technique proved to be faster than the conventional technique at all stages, with statistically significant differences (Table 3).

**Table 3:** Time recorded per second during conventional and digital impression-taking N: number; Conv: conventional; Dig: digital; max: maxillary; mand: mandibular

		Average duration (seconds)	Standard deviation	Mean difference Conv-Dig	P (paired Student's t-test)	Effect size
Preparatory time	Conv max	44.6	11.44	22.8	p<0.05	1.72
	Dig max	21.8	5.45			
	Conv mand	37	10.04	15.2	p<0.05	1.37
	Dig mand	21.8	5.45			
Working time	Conv max	123.6	22.27	63.3	p<0.05	2.21
	Dig max	60.3	14.56			
	Conv mand	120.4	18.04	47.4	p<0.05	2.04
	Dig mand	73	19.10			
Total time	Conv	326	35	175	p<0.05	3.90
	Dig	151	27			

Around 62.5% of participants preferred the comfort of digital impression, with no significant correlation between age or gender and this perception. Perception of fatigue was significantly associated with age; 60% of patients aged 10.02(±1.95) found the conventional technique more tiring and 17.5% aged 8.43(±2.70) for digital impressions. Gender significantly influenced the perception of the duration of the technique; 62.5% of girls found the conventional technique longer, compared to 58% of boys. Older patients (mean: 9.88 years) preferred digital impressions, while younger ones (mean: 8.03 years) preferred the accessories of the conventional method, with significant differences between the mean ages (p=0.031). Finally, the order of impression-taking influenced the perception of fatigue (p=0.045) (Table 4).

**Table 4:** Distribution of patient preferences for the two impression techniques according to sociodemographic characteristics (age and gender) Conv: conventional; Dig: digital

					Conv	Dig	No preference	Don't know	p
Most comfortable technique	N total (%)				13 (32.5%)	25 (62.5%)	2 (5%)	0 (0%)	-
	Gender		F		6 (38%)	9 (56%)	1 (6%)	0 (0%)	0.866
			M		7 (29%)	16 (67%)	1 (4%)	0 (0%)	
	Age (year)				8.27±1.91	9.68±2.19	11.00±4.24	-	0.317
Most tiring technique	N total (%)				24 (60%)	7 (17.5%)	9 (22.5%)	0 (0%)	-
	Gender			F	10 (62.5%)	2 (12.5%)	4 (25%)	0 (0%)	0.817
				M	14 (58%)	5 (21%)	5 (21%)	0 (0%)	
	Age (year)				10.02±1.95	8.43±2.70	8.00±2.14	-	0.019
Technique using preferred accessories	N total (%)				16 (40%)	23 (57.5%)	1 (2.5%)	0 (0%)	-
	Gender	F			8 (50%)	8 (50%)	0 (0%)	0 (0%)	0.601
		M			8 (33%)	15 (63%)	1 (4%)	0 (0%)	
	Age (year)				8.03±1.70	10.28±2.15	6.5	-	0.004
Most stressful technique	N total (%)				8 (20%)	12 (30%)	19 (47.5%)	1 (2.5%)	-
	Gender	F			5 (31%)	4 (25%)	6 (38%)	1 (6%)	0.285
		M			3 (13%)	8 (33%)	13 (54%)	0 (0%)	
	Age (year)				10.38±2.26	9.58±2.39	8.74±2.14	7.5	0.231
Technique that took the most time	N total (%)				24 (60%)	12 (30%)	1 (2.5%)	3 (7.5%)	-
	Gender	F			10 (62.5%)	2 (12.5%)	1 (6%)	3 (19%)	0.022
		M			14 (58%)	10 (42%)	0 (0%)	0 (0%)	
	Age (year)				9.85±2.263	9.04±2.005	7.00	6.50±0.866	0.032
Most playful technique	N total (%)				19 (47.5%)	20 (50%)	1 (2.5%)	0 (0%)	-
	Gender	F			8 (50%)	8 (50%)	0 (0%)	0 (0%)	1.000
		M			11 (46%)	12 (50%)	1 (4%)	0 (0%)	
	Age (year)				9.08±2.33	9.55±2.28	8.00	-	0.659
Preferred technique for new impression	N total (%)				15 (37.5%)	25 (62.5%)	0 (0%)	0 (0%)	-
	Gender	F			6 (37.5%)	10 (62.5%)	0 (0%)	0 (0%)	1.000
		M			9 (37.5%)	15 (62.5%)	0 (0%)	0 (0%)	
	Age (year)				8.30±1.78	9.88±2.35	-	-	0.031

## Discussion

Our study aimed to compare young patients' perceptions of digital impression techniques using Trios 3 (3Shape) and conventional alginate impressions to determine the most comfortable and convenient method. It should be noted that this comparison had never been performed before at the Dental Consultation and Treatment Center in Casablanca or in Morocco.

Yilmaz and Aydin [15] compared digital impressions using the 3Shape system with conventional impressions in 30 children. The digital impression method, compared with the conventional impression method, was found to be both more comfortable and preferable by the children, but there was no difference in terms of the time required to take impressions. Bosoni et al. [16] assessed use in 24 orthodontic patients aged 6-11 years. Digital impression is preferred by children, and it is significantly faster and more comfortable than conventional alginate impression.

Glisic et al. [18] evaluated Trios Classic (earlier version) in 59 children and adolescents. Children preferred the intraoral scan rather than the alginate impression. Chairside time was equal for the two procedures, as were the measurements of maxillary dental arch distances. The two procedures were equal in cost at 3.6 years. Burhardt et al. [21] used a different system, including Lava COS (3M ESPE, Saint Paul, Minnesota, United States) and Cerec Omnicam (Dentsply Sirona, Charlotte, North Carolina, United States), in 38 children. Young orthodontic patients preferred the digital impression techniques over the alginate method, although alginate impressions required the shortest chairside time.

The choice of the 6-14-year age group is justified by the specific characteristics of this period, marked by active dentomaxillary development (eruption of permanent teeth) and notable behavioral characteristics (fear, stress, potential agitation). A minimum age of six generally provides a sufficient level of understanding and communication skills to facilitate interaction and cooperation during dental procedures.

Based on experimental protocols developed in previous studies for sample size selection, our work is in line with recent research on an age group that has been little explored in the scientific literature. The mean age in our study was 9.3±2.25 years, which is comparable to that reported by Bosoni et al. [16] (8.8 years; SD=1) and Yilmaz and Aydin [15] (10.16 years; SD=1.77).

A non-probability convenience sample was chosen for practical reasons, recruiting patients from the waiting room. We opted for a seven-day interval between the two impression techniques in order to avoid influencing the children in their choice of technique, to avoid tiring them by taking two successive impressions, and to minimize memory bias in them, given that both types of impressions were taken from the same patient. Although this sample is not entirely representative of all children attending the dental consultation and treatment center, this does not affect the validity of the results of our semi-experimental comparative study.

Customized questionnaires, inspired by previous work (Yilmaz and Aydin [15], Bosoni et al. [16]), were used to assess patient comfort, cooperation, time required, and preferences for the two impression techniques. These questionnaires were translated into the Arabic dialect to meet the needs of the study.

The results show that digital impressions are perceived as more comfortable than alginate impressions by 62.5% of patients. Age had no significant impact on the perception of comfort, contrary to what was reported by Burzynski et al. [22], who suggest that age may influence patient preferences. No significant difference was observed between genders, with results similar to those of Serrano-Velasco et al. [14]. This result can be explained by the fact that the Trios 3 (3Shape) intraoral camera is smaller than the impression tray, which avoids stretching the lips or cheeks and thus minimizes discomfort. Burzynski et al. [22] showed similar results regarding the use of another intraoral scanner, iTero (Align, San Jose, California, United States), which did not cause pain or discomfort, thanks to its small size and less bulky wand. No significant difference was observed between digital and conventional impressions regarding hand/foot movements, which differs from the results of Yilmaz and Aydin [15] (p=0.01), who attributed the movements to discomfort caused by alginate.

Respiratory problems

No respiratory problems were reported during digital impression. However, 20% of patients experienced breathing difficulties with the alginate impression in the maxilla, with a significant difference observed between the maxilla and mandible (p=0.008). These findings are consistent with those of Bosoni et al. [16], who found that digital impressions were considered less invasive in terms of breathing difficulties. The breathing difficulties associated with alginate impressions may be due to the soft consistency of the material, which can move towards the pharynx and block the airways under the effect of gravity.

Gag reflex

During conventional maxillary impression-taking sessions, 27.5% of children experienced a gag reflex, compared to only 2.5% of patients who experienced this discomfort during digital impression-taking, and the difference observed was significant. A significant difference was also observed between conventional maxillary impressions and mandibular impressions (p=0.002). The same observation was made by Glisic et al. [18]. This gag reflex can be triggered by tactile stimuli in the oral cavity or pharynx, such as the use of a bulky impression tray or contact of the impression material with reflexogenic areas such as the soft palate and the back of the tongue.

Level of cooperation: behavior and anxiety

Taking impressions in children can be challenging due to their varying behavior, particularly between different age groups. In our study, the experienced operator, who had taken more than 100 impressions, demonstrated kindness and attentiveness and provided clear explanations, which helped to promote positive cooperation during the impression-taking sessions.

Statistical tests revealed that age and gender did not significantly influence children's behavior during either digital or conventional impressions. The sequence of impression-taking could have an impact on children's behavior, and in our study, it was standardized: mandibular impression followed by maxillary impression, in accordance with reference studies to avoid bias and facilitate comparison of results. This did not prevent some children from reporting that conventional maxillary impression-taking was suffocating and unpleasant.

The results show that children experienced more anxiety with conventional impression-taking (40% feeling uncomfortable) than with digital impression-taking (22.5%), with no statistically significant difference between the two techniques. These results are consistent with the studies by Sfondrini et al. [8] and Glisic et al. [18], who used similar materials and scanners.

Time required for impression-taking

Our study reveals a significant difference in terms of time efficiency between conventional and digital impression techniques, with digital impressions being significantly faster. Digital impression preparation took an average of 17.7 seconds, compared to 81.5 seconds for conventional impressions. Similarly, the impression-taking process was faster with digital impressions, taking 60.3 seconds for the maxilla and 73 seconds for the mandible, compared to 123.6 seconds and 120.4 seconds for conventional impressions, respectively. These results are consistent with several previous studies [6,15,16]. However, some studies have reported contrasting results. Burhardt et al. [21] reported that conventional impressions were faster due to the slower speed of the Lava COS scanner. Similarly, Burzynski et al. [22] found that conventional impressions (6.4 minutes) were faster than digital impressions taken with iTero (7 minutes) and Trios Color (8.6 minutes) scanners, highlighting the impact of scanner type and speed on impression time.

The duration of the impression-taking process is influenced by various factors, including the scanner model and capture speed. For example, the Trios 3 3Shape scanner captures 1,875 images per second, while the Trios 5 Wireless captures 2,400, offering a superior 3D model quality. Other factors, such as powder use, mouth opening extent, and the presence or absence of the second permanent molar, can also impact acquisition time. Overall, the continuous evolution of scanners has led to improved efficiency and accuracy in digital impression-taking [23,24].

The next two tables compare time requirements for digital impression systems and traditional impression-taking methods. Table 5 focuses on scanning and processing times for digital systems, while Table 6 examines the impression-taking times for traditional and digital materials [24].

**Table 5:** Comparison of the time required for scanning and file processing of different digital impression systems

Model	Cerec Omnicam (Dentsply Sirona, Charlotte, North Carolina, United States)	Trios Color (3Shape, Copenhagen, Denmark)	CS 3600 (Carestream, Rochester, New York, USA)	CondorScan (Biotech, France)	DWIO (Dental Wings, Quebec, Canada)	iTero Element (Align, San Jose, California, United States)
Scanning time full arch/min	4	3	5	3	5 (powder-free)	2-3
File processing time	2 minutes	2 minutes	1 minute	30 seconds	Instant result	Instant result

**Table 6:** Comparison of the time required for impression-taking: traditional materials versus digital impressions

Traditional materials	Alginate (fast set)	Reversible hydrocolloid	Polysulfide	Polyether	Condensation silicone	Addition silicone
Setting time in minutes	2-3	Variable	8-10	7-9	6-8	6-8
Digital impression systems	iTero (Align, San Jose, California, United States)	Trios (3Shape, Copenhagen, Denmark)	Cerec Omnicam (Dentsply Sirona, Charlotte, North Carolina, United States)	Lava COS (3M ESPE, Saint Paul, Minnesota, United States)	Medit (Medit Corp., South Korea)	
Acquisition time in minutes	2-5	2-4	3-6	2-5	2-4	

Patient preferences

Our results indicate that 62.5% of children perceive the digital impression technique as more comfortable than conventional impressions, which is consistent with the findings of several previous studies [6,14-16,21]. However, Grünheid et al. [25] reported contrasting results, with 73.3% of patients preferring alginate impressions due to the discomfort caused by the large size of the intraoral camera used (Lava COS). In terms of efficiency, 60% of the children in our study felt that conventional impressions took longer than digital impressions, confirming the advantages of the digital technique and corresponding to the results of several studies [6,15,16,21]. In terms of stress levels, 30% of children found digital impressions more stressful, while 47.5% had no preference, probably due to the difficulty of understanding the concept of stress and sometimes confusing it with fear.

In our study, some children attributed their stress to fear of swallowing the alginate material and choking. The digital impression technique was generally better tolerated due to its speed and less invasive nature, and the children's anxiety subsided after a clear explanation of both techniques.

When asked which technique they liked best, the results were almost evenly split, with 50% preferring digital impressions and 47.5% preferring the conventional impressions. The reasons for their preferences varied, with children who preferred the conventional impressions citing the colorful impression trays, the fruity taste of the alginate, and the malleable texture, which reminded them of a sensory activity. In contrast, children who preferred the digital impression enjoyed seeing their teeth on a screen, manipulating and zooming in on images, which they compared to an interactive video game.

It should be noted that 60% of the children participating in our clinical trial found conventional impressions more tiring, compared to only 17.5% for digital impressions. According to the children, the fatigue associated with conventional impressions was mainly due to the prolonged duration of the procedure, while for digital impressions, it was the requirement to keep their mouths open during the scan that caused fatigue. In contrast, Glisic et al. [18] suggest that intraoral scanners can help minimize fatigue in children by allowing them to take breaks between scan sequences.

About 57% of children preferred the accessories of the digital impression, citing the interactive screen and intraoral camera as highlights, with some even comparing the camera to a "magic wand". In contrast, 40% preferred the conventional impression, appreciating the shape and colors of the impression trays and the alginate mixing process, which they likened to baking.

Ultimately, 62.5% of children chose the digital impression as their preferred method for future impressions, while 37.5% opted for the conventional impression. Although some children had apprehensions about the digital impression, such as the noise and appearance of the intraoral scanner, the trend towards preferring digital impressions is consistent with previous studies, which have reported preference rates ranging from 63.3% to 100% [3,6,9,13].

Despite its advantages, the digital impression technique presents challenges, including a longer learning curve and high initial costs (the digital procedure is initially 10.7 times more expensive than the conventional method; however, the cost balances out after 3.6 years of use [14,18]). Alginate impressions remain relevant in certain situations, such as removable prostheses or children who have difficulty staying still or who salivate excessively [26]. The psychological approach and practitioner experience are crucial for optimal results, regardless of the technique used.

Study's strengths

This study investigates digital and conventional impression techniques in children aged 6-14 years, an age group that is relatively underexplored and has not yet been studied at the national level. The work aims to fill a gap in the literature by providing a comprehensive comparison of accuracy, patient comfort, and practical implications across a broad pediatric age range.

Our study benefits from a controlled experimental design with randomization to minimize bias and examines the impact of factors such as age, sex, and order of impression-taking on children's perception, thus bringing a new perspective on the experience of young patients.

Study's limitations

The difficulties encountered during the study include problems with patient recruitment, which required a change in sampling method, moving from simple random sampling to a rapid convenience sample due to some parents refusing to continue impression-taking sessions for reasons such as availability, residence distance, and child illness.

Moreover, some children needed additional explanations to comprehend and complete the questionnaire. The use of a single type of impression material and a single intraoral scanner could limit the generalization of results.

## Conclusions

This study rejected the null hypothesis, demonstrating the efficiency and benefits of digital impressions over conventional ones in terms of comfort and speed, while cooperation and anxiety levels were similar for both techniques. Digital impressions were notably preferred by children, with many expressing a desire to use this technique again in the future. The results, consistent with previous studies, suggest that intraoral scanners offer advantages for children, particularly older ones who can remain still and manage saliva control. Future research directions could include assessing perceptions in younger children or those with disabilities, evaluating costs, comparing optical scanners, and assessing digital model accuracy. By exploring these areas, we can continue to improve our practices and provide optimal care for our patients.
